# Association of Family Income and Risk of Food Insecurity With Iron Status in Young Children

**DOI:** 10.1001/jamanetworkopen.2020.8603

**Published:** 2020-07-30

**Authors:** Imaan Bayoumi, Patricia C. Parkin, Catherine S. Birken, Jonathon L. Maguire, Cornelia M. Borkhoff

**Affiliations:** 1 Department of Family Medicine, Queen’s University, Kingston, Ontario, Canada; 2 Centre for Studies in Primary Care, Queen’s University, Kingston, Ontario, Canada; 3 Division of Paediatric Medicine and the Paediatric Outcomes Research Team (PORT), The Hospital for Sick Children, Toronto, Ontario, Canada; 4 Institute of Health Policy, Management, and Evaluation, Dalla Lana School of Public Health, University of Toronto, Toronto, Ontario, Canada; 5 Department of Pediatrics, Faculty of Medicine, University of Toronto, Toronto, Ontario, Canada; 6 Child Health Evaluative Sciences, Sick Kids Research Institute, Toronto, Ontario, Canada; 7 The Applied Health Research Centre, Li Ka Shing Knowledge Institute, Toronto, Ontario, Canada; 8 St Michael’s Hospital, Toronto, Ontario, Canada

## Abstract

**Importance:**

Iron deficiency (ID) has the greatest prevalence in early childhood and has been associated with poor developmental outcomes. Previous research examining associations of income and food insecurity (FI) with ID is inconsistent.

**Objective:**

To examine the association of family income and family risk of FI with iron status in healthy young children attending primary care.

**Design, Setting, and Participants:**

This cross-sectional study included 1245 children aged 12 to 29 months who attended scheduled primary care supervision visits from 2008 to 2018 in Toronto, Canada, and the surrounding area.

**Exposures:**

Family income and risk of FI were collected from parent-reported questionnaires. Children whose parents provided an affirmative response to the 1-item FI screen on the Nutrition Screening Tool for Every Toddler or at least 1 item on the 2-item Hunger Vital Sign FI screening tool were categorized as having family risk of FI.

**Main Outcomes and Measures:**

Iron deficiency (serum ferritin level <12 ng/mL) and ID anemia (IDA; serum ferritin level <12 ng/mL and hemoglobin level <11.0 g/dL). All models were adjusted for age, sex, birth weight, body mass index *z *score, C-reactive protein level, maternal education, breastfeeding duration, bottle use, cow’s milk intake, and formula feeding in the first year.

**Results:**

Of 1245 children (595 [47.8%] girls; median [interquartile range] age, 18.1 [13.3-24.0] months), 131 (10.5%) were from households with a family income of less than CAD $40 000 ($29 534), 77 (6.2%) were from families at risk of FI, 185 (14.9%) had ID, and 58 (5.3%) had IDA. The odds of children with a family income of less than CAD $40 000 having ID and IDA were 3 times higher than those of children in the highest family income group (ID: odds ratio [OR], 3.08; 95% CI, 1.66-5.72; *P* < .001; IDA: OR, 3.28; 95% CI, 1.22-8.87; *P* = .02). Being in a family at risk of FI, compared with all other children, was not associated with ID or IDA (ID: OR, 0.43; 95% CI, 0.18-1.02; *P* = .06; IDA: OR, 0.16; 95% CI, 0.02-1.23; *P* = .08).

**Conclusions and Relevance:**

In this study, low family income was associated with increased risk of ID and IDA in young children. Risk of FI was not a risk factor for ID or IDA. These findings suggest that targeting income security may be more effective than targeting access to food to reduce health inequities in the prevention of iron deficiency.

## Key Points

**Question:**

Are family income and risk of food insecurity associated with iron status in healthy young children?

**Findings:**

In this cross-sectional study of 1245 children aged 12 to 29 months, those with a family income of less than CAD $40 000 had 3 times higher odds of having iron deficiency and iron deficiency anemia than children in the highest family income group. Risk of food insecurity was not associated with iron deficiency or iron deficiency anemia.

**Meaning:**

The findings of this study suggest that low family income may be an important risk factor for iron deficiency and iron deficiency anemia, but family risk of food insecurity is not.

## Teaser

This cross-sectional study examines the association of family income and family risk of food insecurity with iron status in healthy young children attending primary care in Toronto, Canada.

## Introduction

Iron is an essential nutrient, and iron deficiency (ID) in early life has been associated with potentially irreversible poor developmental outcomes.^1,2,3,4,5,6^ The prevalence of ID in childhood peaks between age 12 and 24 months^7^ because of rapid growth, depletion of prenatally acquired iron stores, and the transition to complementary foods. In high-income countries, an estimated 9% to 12% of young children have nonanemic ID and 1% to 3% have ID anemia (IDA).^8,9,10^ Optimizing iron status in early childhood may represent an opportunity to address a modifiable factor to improve young children’s health.

Low family income is an important social determinant of children’s health and has been associated with profound negative consequences on child health, particularly when the exposure to poverty is prolonged or occurs in early childhood.^11,12,13^ Child poverty remains prevalent, affecting 16.2% of US children.^14^ Family income affects children’s growth and development,^12^ including nutritional, mental, and developmental health.^15,16^

Food security is defined as the state “when all people, at all times, have physical, social, and economic access to sufficient, safe, and nutritious food which meets their dietary needs and food preferences for an active and healthy life.”^17^ An estimated 12% of US households reported experiencing some level of food insecurity (FI) in the preceding 12 months, including 16% of households with children under the age of 18 years.^18^ Household FI has been associated with poor child health outcomes,^19^ adversely affecting developmental health,^20,21^ children’s mental health,^22,23^ and parental mental health.^24^

The American Academy of Pediatrics recommends screening for ID using risk assessment (including income)^10^ and screening for FI.^25^ It is unclear whether family income or FI are independently associated with ID or whether primary care screening should focus on 1 or both risk factors. Previous studies have examined the association between FI and iron status^26,27,28,29,30^ or income and ID^9,31,32^ in children residing in high-income countries with inconsistent results. The few studies accounting for both household income and FI are now dated, were conducted in high-risk populations, assessed varying levels of FI severity, or did not adjust for potential confounders, such as feeding practices.^9,28^

Understanding the association of family income and family risk of FI with iron status in healthy young children attending primary care may help to inform guidelines for screening for ID as well as policy approaches aimed at reducing health inequities in the prevention of ID. The objective of this study was to examine the association of family income and family risk of FI with iron status in healthy young children attending primary care while adjusting for child characteristics and feeding practices.

## Methods

### Participants

This was a cross-sectional study of healthy urban children who attended scheduled health supervision visits at The Applied Research Group for Kids (TARGet Kids!) participating pediatric or family medicine primary care practices between December 2008 and January 2018. TARGet Kids! is a primary care practice–based research network based in Toronto, Canada. TARGet Kids! is an ongoing, open, longitudinal cohort enrolling healthy children from birth to age 5 years. The profile of this cohort has been previously described.^33^ Study participants were recruited by trained research personnel embedded in participating practices. Parents completed a standardized questionnaire based on the Canadian Community Health Survey addressing child and family characteristics, nutrition, and feeding practices. Parents also completed the Nutrition Screening Tool for Every Toddler (NutriSTEP) questionnaire, a validated parent-completed measure of nutritional risk.^34^ These questionnaires included questions regarding family income and risk of FI. Blood samples (for serum ferritin, hemoglobin, and C-reactive protein [CRP] levels) and anthropometric measures were also collected at these visits.

TARGet Kids! exclusion criteria at enrollment are as follows: genetic or chronic health conditions (except asthma); severe developmental delay; gestational age less than 32 weeks; acute illness; and parents unable to communicate in English.^33^ For the purpose of this study, children were included if they were aged 12 to 29 months (when children have the highest prevalence of iron deficiency^7^ and attend health supervision visits scheduled at ages 12, 15, 18, and 24 months) and had complete data on exposure and outcome variables, including parent-reported family income and family risk of FI, serum ferritin level, and CRP level. If a child’s record contained data from multiple visits, the first visit with serum ferritin testing was used for analysis. A child with an affirmative response for family at risk of FI at any visit during this short interval was considered to be at risk of FI. Children with serum ferritin levels greater than 200 ng/dL (to convert to micrograms per liter, multiply by 1.0) were excluded, given that this was beyond the upper limit of the reference interval.^35^ Children with CRP levels greater than 0.5 mg/dL (to convert to milligrams per liter, multiply by 10), suggesting acute systemic inflammation, were excluded because serum ferritin can be falsely elevated in this context.^10,36^ Children taking iron supplementation or a multivitamin containing iron were excluded.

Consent was obtained from parents of participating children, and ethics approval was granted by the Research Ethics Boards at the Hospital for Sick Children and St Michael’s Hospital. This report followed the Strengthening the Reporting of Observational Studies in Epidemiology (STROBE) reporting guideline.

### Exposure Variables

We used 2 exposure variables for analyses, ie, parent-reported family income and parent-reported family risk of FI. The questions, response options, and definition of an affirmative FI response and family income categories are shown in Table 1.

**Table 1.** Exposure Variables

Exposure	Question	Response options	Affirmative response
Family income	What was your total family income before taxes last year?^a^	<$40 000	NA
		$40 000-$79 999	
		$80 000-$149 999	
		≥$150 000	
Family at risk for food insecurity	1-item NutriSTEP question, “I have difficulty buying food I want to feed my children because food is expensive”	Most of the time	Most of the time or sometimes
		Sometimes	
		Rarely	
		Never	
	2-item Hunger Vital Sign questions, “Within the past 12 mo, we worried about whether our food would run out before we got money to buy more,” and “Within the past 12 mo, the food we bought just didn’t last and we didn’t have money to buy more”	Often true	Often true or sometimes true
		Sometimes true	
		Never true	

Abbreviation: NA, not applicable.

^a^ To convert Canadian dollars to US dollars, multiply by 0.74.

Family income was collected in 4 categories, with the lowest income category and the lower middle income category approximating the low income cutoff (CAD $44 266 [$32 684] for a 4-person household) and the median family income (CAD $82 859 [$61 180] for a 4-person household), respectively, for Toronto, Canada.^37^

Family risk of FI was collected using 2 approaches, as follows: a 1-item question used to screen for risk of FI from the NutriSTEP questionnaire^34^ (available from 2008-2018) and the Hunger Vital Sign, a 2-item FI screening tool^38^ (available from 2013-2018). The Hunger Vital Sign is based on the 18-item US Household Food Security Survey Module (HFSSM),^39^ and an affirmative response to either of the 2 items has a sensitivity of 97% and specificity of 83% for identifying families at risk of FI, using the 18-item HFSSM as the criterion measure.^40^ Our group previously validated the 1-item NutriSTEP question using the 2-item Hunger Vital Sign as the criterion measure.^41^ We found the NutriSTEP question had a sensitivity of 85% and a specificity of 91%. Family at risk of FI was defined as an affirmative response to the 1-item NutriSTEP question (“most of the time” or “sometimes”) or to 1 or both of the Hunger Vital Sign questions (“often true” or “sometimes true”) (Table 1).

### Outcome Variables

The primary outcome was ID defined as serum ferritin of less than 12 ng/dL, as recommended by the World Health Organization and the American Academy of Pediatrics for this age group.^10,42^ Serum ferritin has been described as the best indicator of iron stores in the absence of inflammation.^43^ To account for acute systemic inflammation, children with CRP levels greater than 0.5 mg/dL were excluded, and CRP was included as a covariate.^36,44^ Blood samples were refrigerated and transported to the laboratory the same day. The secondary outcome was IDA, defined as serum ferritin levels of less than 12 ng/dL and hemoglobin levels of less than 11.0 g/dL (to convert to grams per liter, multiply by 10).^35^

### Other Variables

Covariates that might influence serum ferritin level, family income, or risk of FI (based on peer-reviewed literature), were identified a priori, including child age; child sex; birth weight; sex- and age-adjusted body mass index (BMI) *z* score; CRP level; maternal education; infant feeding in the first year of life; breastfeeding duration; bottle use after age 15 months; and daily cow’s milk intake.^45,46,47,48^ Maternal education was determined based on parent-reported highest level of educational attainment. Infant feeding in the first year of life was determined from response to the question, “Which scenario best describes your child in the first year of life? (1) My child received infant formula 80% to 100% of the time; (2) My child received breast milk 80% to 100% of the time; (3) My child received both breast milk and formula equally.” Breastfeeding duration was determined based on parental recall, considered valid and reliable in the child’s first 3 years of life.^44^ Children who were never breastfed were assigned a breastfeeding duration of 0 months, and those currently breastfeeding were defined as having a breastfeeding duration equal to the child’s current age in months.

### Statistical Analysis

Descriptive statistics of the exposures, outcomes, and covariates were calculated for the entire sample, as well as for each of the 4 family income categories (<CAD $40 000 [$29 534], CAD $40 000-$79 999 [$29 534-$59 068], CAD $80 000-$149 999 [$[59 068-$110 752], and ≥CAD $150 000 [$110 753]) and 2 family at risk of FI categories (yes and all other). To assess the association of family income and family risk of FI with ID, we examined univariate associations between each of the exposure variables and ID and IDA. Then, 2 multivariable logistic regression models were constructed, the first to evaluate the association between family income and ID (model 1a) and the second to evaluate the association between family risk of FI and ID (model 1b). Models 1a and 1b were compared with the fully adjusted model, which included family income and family risk for FI (model 2).

We used similar models to evaluate the association between our exposure variables and our secondary outcome, IDA. All models were adjusted for all covariates previously described regardless of statistical significance.^49^ We used restricted cubic spline transformation to test the assumption of a linear association between age and serum ferritin; after nonlinearity was confirmed by Loess curves, splines were used to model the association of age. We selected 3 knot points to correspond to the age at scheduled visits (ie, 15, 18, and 24 months); these knot locations were selected at scheduled visit times, when data points were clustered.^7^

A likelihood ratio test between the main effects model and the model with the interaction term (family income × family risk of FI) yielded a *P* = .59, suggesting that we could rule out the possibility of an interaction between the 2 exposure variables. Therefore, no interaction term was included in any model. Multicollinearity was assessed using the variance inflation factor.^50^ All variance inflation factors were less than 2, suggesting that multicollinearity was unlikely. The maximum rate of missing data for any covariate was 8%. Multiple imputation using the fully conditional specification method was used to impute missing covariates.^51^ To reduce the potential for bias, models were run on 20 imputed data sets using all identified covariates.^52^ Results of the 20 imputed data sets were combined, and the parameter estimates (95% CIs) for the adjusted pooled models were reported. Sensitivity analyses were performed, including children with a response of “rarely” to the 1-item NutriSTEP question in our definition of a family at risk of FI. Statistical significance was defined as *P* < .05, and all statistical tests were 2-sided. Statistical analysis was conducted using SAS version 9.4 (SAS Institute).

## Results

A total of 4571 children were recruited to participate; there were 1446 children with available family income, family at risk of FI, and serum ferritin data. Of these, 201 were excluded, as shown in eBox 1 in the Supplement. Previous TARGet Kids! research found that children with and without blood testing had similar demographic characteristics,^47^ which we also observed (eBox 2 in the Supplement).

The final study sample of 1245 children included 595 (47.8%) girls, and the median (interquartile range) age was 18.1 (13.3-24.0) months. Descriptive statistics are shown in Table 2, which includes a total column showing the distribution of participant characteristics for the final analytic sample (with imputation), demonstrating minimal selection bias owing to missing data.^53^

**Table 2.** Characteristics of 1245 Study Participants

Characteristic	Response sample, No. (%)	Imputed sample, No. (%)^a^
**Patient-level characteristics**		
Female sex (n = 1245)	595 (47.8)	595 (47.8)
Child’s age, median (IQR), mo (n = 1245)	18.1 (13.3 to 24.0)	18.1 (13.3 to 24.0)
Birth weight, median (IQR), kg (n = 1154)	3.3 (2.9 to 3.7)	3.3 (2.9 to 3.7)
BMI *z *score (n = 1220)		
Median (IQR)	0.07 (−0.64 to 0.82)	0.06 (−0.64 to 0.82)
<−2	32 (2.6)	32 (2.6)
−2 to ≤1	947 (77.6)	969 (77.8)
1 to ≤2	184 (15.1)	187 (15.0)
2 to ≤3	46 (3.8)	46 (3.7)
>3	11 (0.9)	11 (0.9)
Maternal ethnicity^b^ (n = 1127)		
European	733 (65.0)	817 (65.6)
Asian	235 (20.9)	258 (20.7)
Other	159 (14.1)	170 (13.7)
Maternal education (n = 1217)		
University	984 (80.9)	1005 (80.7)
College/trades certificate	137 (11.3)	144 (11.6)
High school or less	96 (7.9)	96 (7.7)
Family income, CAD$ (n = 1245)^c^		
≥150 000	517 (41.5)	517 (41.5)
80 000-149 999	435 (34.9)	435 (34.9)
40 000-79 999	162 (13.0)	162 (13.0)
<40 000	131 (10.5)	131 (10.5)
Family at risk for food insecurity (n = 1245)		
All other	1168 (93.8)	1168 (93.8)
Yes	77 (6.2)	77 (6.2)
**Infant feeding practices**		
Infant feeding first year of life (n = 1221)		
Mostly formula fed	237 (19.4)	242 (19.4)
Mostly breastfed	765 (62.7)	778 (62.5)
Breast milk and formula equally	219 (17.9)	225 (18.1)
Breastfeeding duration, mo (n = 1203)		
<12	530 (44.1)	554 (44.5)
≥12	673 (55.9)	691 (55.5)
Bottle use >15 mo (n = 1150)		
No	730 (63.5)	789 (63.4)
Yes	420 (36.5)	456 (36.6)
Daily cow’s milk intake (n = 1207)		
≤2 cups	876 (72.6)	908 (72.9)
>2 cups	331 (27.4)	337 (27.1)
**Laboratory characteristics**		
Serum ferritin, median (IQR), ng/dL (n = 1245)	23 (15 to 35)	23 (15 to 35)
Hemoglobin, median (IQR), g/dL (n = 1090)^d^	11.8 (11.2 to 12.4)	11.8 (11.2 to 12.4)
Iron deficiency (n = 1245)	185 (14.9)	185 (14.9)
Iron deficiency anemia (n = 1090)^d^	58 (5.3)	58 (5.3)
CRP, mg/dL (n = 1245)		
≤0.1	1061 (85.2)	1061 (85.2)
>0.1 to ≤0.5	184 (14.8)	184 (14.8)

Abbreviations: BMI, body mass index; CRP, C-reactive protein; IQR, interquartile range.

SI conversion factors: To convert CRP to milligrams per liter, multiply by 10; hemoglobin to grams per liter, multiply by 10; serum ferritin to micrograms per liter, multiply by 1.0.

^a^ Based on the final analytic sample. Data are for 1 imputed data set.

^b^ European includes western European, eastern European, Australian, or New Zealander; Asian includes east Asian, southeast Asian, south Asian, west Asian; and other includes African, Caribbean, Latin American, and North American Indigenous, and 2 or more ethnic groups.

^c^ To convert Canadian dollars to US dollars, multiply by 0.74.

^d^ Based on the 1090 children with complete data for iron deficiency anemia.

Table 3 shows participant characteristics for the final analytic sample by exposure variables (ie, family income category and family at risk of FI). Notably, 34 of 131 children (26.0%) from households with a family income of less than CAD $40 000 ($29 534) were iron deficient; however, 7 of 77 children (9.1%) in families at risk of FI were iron deficient compared with 178 of 1168 children (15.2%) from all other FI categories. There was an income gradient for the variable family at risk for FI, ranging from 34 children (26.0%) living in the lowest-income families to 5 of 517 children (1.0%) living in the highest-income families. There was some variation in the distribution of covariates. Compared with children living in the highest-income families, those in the lowest-income families had higher body mass index *z *scores (≥2, 21 [4.1%] vs 9 [6.9%]), fewer were breastfed after age 12 months (294 [56.9%] vs 54 [41.2%]), more were mostly formula fed in the first year of life (59 [11.4%] vs 49 [37.4%]), more had cow’s milk intake greater than 2 cups per day (131 [25.3%] vs 60 [45.8%]), more used bottles after age 15 months (160 [31.0%] vs 75 [57.3%]), and more had mothers with a high school diploma or less (15 [2.9%] vs 48 [36.6%]). More children in families at risk of FI compared with all other children were mostly formula fed (28 [36.4%] vs 214 [18.3%]), and fewer were breastfed after age 12 months (30 [39.0%] vs 661 [56.6%]); however, there were no differences between children in families at risk of FI compared with all other children in cow’s milk intake greater than 2 cups per day, bottle use after age 15 months, and maternal educational attainment.

**Table 3.** Characteristics of Study Cohort by Family Income Category and Family at Risk of Food Insecurity^a^

Characteristic	No. (%)					
	Family income, CAD$^b^				Family at risk of food insecurity	
	<40 000 (n = 131)	40 000-79 999 (n = 162)	80 000-149 999 (n = 435)	≥150 000 (n = 517)	Yes (n = 77)	All other (n = 1168)
**Patient-level characteristics**						
Female sex	68 (51.9)	90 (55.6)	208 (47.8)	229 (44.3)	39 (50.7)	556 (47.6)
Child’s age, median (IQR), mo	18.4 (15.1 to 24.1)	18.1 (14.0 to 24.0)	18.1 (12.9 to 24.0)	18.1 (12.9 to 24.0)	18.2 (15.0 to 24.0)	18.1 (13.2 to 24.0)
Birth weight, median (IQR), kg	3.2 (2.7 to 3.6)	3.3 (2.8 to 3.6)	3.3 (2.9 to 3.7)	3.4 (3.0 to 3.7)	3.2 (2.6 to 3.6)	3.3 (2.9 to 3.7)
BMI *z *score						
Median (IQR)	0.26 (−0.56 to 1.00)	0.04 (−0.69 to 0.72)	0.00 (−0.67 to 0.80)	0.11 (−0.65 to 0.82)	0.07 (−0.56 to 0.75)	0.06 (−0.65 to 0.82)
<−2	6 (4.6)	2 (1.2)	13 (3.0)	11 (2.1)	1 (1.3)	31 (2.7)
−2 to ≤1	94 (71.8)	132 (81.5)	341 (78.4)	402 (77.8)	61 (79.2)	908 (77.7)
1 to ≤2	22 (16.8)	22 (13.6)	60 (13.8)	83 (16.1)	12 (15.6)	175 (15.0)
2 to ≤3	5 (3.8)	5 (3.1)	18 (4.1)	18 (3.5)	3 (3.9)	43 (3.7)
>3	4 (3.1)	1 (0.6)	3 (0.7)	3 (0.6)	0 (0)	11 (0.9)
Maternal education						
University	43 (32.8)	113 (69.8)	365 (83.9)	484 (93.6)	43 (55.8)	962 (82.4)
College/trades certificate	40 (30.5)	32 (19.8)	54 (12.4)	18 (3.5)	16 (20.8)	128 (11.0)
High school or less	48 (36.6)	17 (10.5)	16 (3.7)	15 (2.9)	18 (23.4)	78 (6.7)
Family income, CAD$						
≥150 000	0	0	0	517 (100.0)	5 (6.5)	512 (43.8)
80 000-149 999	0	0	435 (100.0)	0	15 (19.5)	420 (36.0)
40 000-79 999	0	162 (100.0)	0	0	23 (29.9)	139 (11.9)
<40 000	131 (100.0)	0	0	0	34 (44.2)	97 (8.3)
Family at risk for food insecurity						
All other	97 (74.0)	139 (85.8)	420 (96.6)	512 (99.0)	0	1168 (100.0)
Yes	34 (26.0)	23 (14.2)	15 (3.4)	5 (1.0)	77 (100.0)	0
**Infant feeding practices**						
Infant feeding first year of life						
Mostly formula fed	49 (37.4)	51 (31.5)	83 (19.1)	59 (11.4)	28 (36.4)	214 (18.3)
Mostly breastfed	45 (34.4)	84 (51.9)	290 (66.7)	359 (69.4)	29 (37.7)	749 (64.1)
Breast milk and formula equally	37 (28.2)	27 (16.6)	62 (14.2)	99 (19.2)	20 (25.9)	205 (17.6)
Breastfeeding duration ≥12 mo	54 (41.2)	84 (51.9)	259 (59.5)	294 (56.9)	30 (39.0)	661 (56.6)
Bottle use >15 mo	75 (57.3)	67 (41.4)	154 (35.4)	160 (31.0)	36 (46.8)	420 (36.0)
Cow’s milk intake >2 cups/d	60 (45.8)	55 (33.9)	91 (20.9)	131 (25.3)	24 (31.2)	313 (26.8)
**Laboratory characteristics**						
Serum ferritin level, median (IQR), ng/dL	22 (11 to 36)	25 (18 to 37)	23 (15 to 33)	22 (15 to 35)	26 (16 to 39)	23 (15 to 34)
Hemoglobin level, median (IQR), g/dL^c^	11.9 (11.2 to 12.6)	11.9 (11.3 to 12.5)	11.8 (11.3 to 12.3)	11.8 (11.2 to 12.3)	11.9 (11.4 to 12.6)	11.8 (11.2 to 12.3)
Iron deficiency	34 (26.0)	17 (10.5)	64 (14.7)	70 (13.5)	7 (9.1)	178 (15.2)
Iron deficiency anemia^c^	12 (10.1)	5 (3.7)	22 (5.7)	19 (4.3)	1 (1.4)	57 (5.6)
CRP level, mg/dL						
≤0.1	120 (91.6)	141 (87.0)	366 (84.1)	434 (83.9)	69 (89.6)	992 (84.9)
>0.1 to ≤0.5	11 (8.4)	21 (13.0)	69 (15.9)	83 (16.1)	8 (10.4)	176 (15.1)

Abbreviations: BMI, body mass index; CRP, C-reactive protein; IQR, interquartile range.

SI conversion factors: To convert CRP to milligrams per liter, multiply by 10; hemoglobin to grams per liter, multiply by 10; serum ferritin to micrograms per liter, multiply by 1.0.

^a^ Based on the final analytic sample. Data are for 1 imputed data set.

^b^ To convert Canadian dollars to US dollars, multiply by 0.74.

^c^ Based on 1090 children with complete data for iron deficiency anemia (family income <CAD $40 000, 119; CAD $40 000-$79 999, 136; CAD $80 000-149 999, 388; ≥CAD $150 000, 447; family at risk of food insecurity, 71; all other, 1019).

In univariate analyses, we found increased odds of ID among children from households with a family income of less than CAD $40 000 ($29 534) compared with children in the highest income category (odds ratio [OR], 2.24; 95% CI, 1.41-3.56; *P* < .001) but not among children from the lower middle income and higher middle income groups. Compared with children in the highest income group, those in the lowest income group also had a higher odds of IDA (OR, 2.53; 95% CI, 1.19-5.37; *P* = .02). Among children in households at risk of FI, we did not find higher odds of ID (OR, 0.56; 95% CI, 0.25-1.23; *P* = .15) or IDA (OR, 0.24; 95% CI, 0.03-1.96; *P* = .16) on univariate analyses.

Table 4 and Table 5 show that after adjusting for age, sex, birth weight, BMI *z *score, CRP level, maternal education, breastfeeding duration, bottle use, daily cow’s milk intake, infant feeding in the first year of life, and family risk of FI, family income of less than CAD $40 000 ($29 534) remained a risk factor for both ID and IDA. The multivariable logistic regression model (model 2) demonstrated that the odds of children from households with a family income of less than CAD $40 000 ($29 534) having iron deficiency was 3.08 times (95% CI, 1.66-5.72; *P* < .001) (Table 4) higher than that for children in the highest family income group, and having IDA was 3.28 times (95% CI, 1.22-8.87; *P* = .02) (Table 5) higher than that for children in the highest family income group.

**Table 4.** Multivariable Logistic Regression Model for the Association of Family Income and Family at Risk for Food Insecurity With Iron Deficiency^a^

Variable	Model 1a		Model 1b		Model 2	
	OR (95% CI)	*P* value	OR (95% CI)	*P* value	OR (95% CI)	*P* value
Child sex						
Male	1 [Reference]	NA	1 [Reference]	NA	1 [Reference]	NA
Female	0.98 (0.70-1.37)	.92	0.97 (0.70-1.36)	.88	0.98 (0.70-1.36)	.89
Child’s age, mo^b^						
12-15	1.40 (1.09-1.79)	.008	1.41 (1.10-1.80)	.007	1.40 (1.09-1.79)	.009
15-18	0.97 (0.80-1.17)	.72	0.96 (0.79-1.16)	.66	0.97 (0.80-1.17)	.73
18-24	1.04 (0.96-1.13)	.33	1.05 (0.97-1.14)	.26	1.05 (0.96-1.14)	.30
24-29	0.80 (0.58-1.09)	.16	0.79 (0.58-1.09)	.15	0.78 (0.56-1.07)	.12
Birth weight	0.74 (0.57-0.97)	.03	0.73 (0.56-0.95)	.01	0.74 (0.57-0.97)	.03
BMI *z* score	1.13 (0.97-1.32)	.12	1.14 (0.97-1.33)	.11	1.13 (0.97-1.32)	.12
CRP level	0.62 (0.45-0.86)	.004	0.61 (0.44-0.84)	.002	0.62 (0.45-0.86)	.004
Infant feeding first year of life						
Mostly breastfed	1 [Reference]	NA	1 [Reference]	NA	1 [Reference]	NA
Breast milk and formula equally	0.37 (0.21-0.66)	<.001	0.43 (0.25-0.75)	.003	0.38 (0.22-0.67)	<.001
Mostly formula fed	0.35 (0.18-0.69)	.003	0.42 (0.22-0.81)	.009	0.36 (0.18-0.70)	.003
Breastfeeding duration, mo						
<12	1 [Reference]	NA	1 [Reference]	NA	1 [Reference]	NA
≥12	2.01 (1.29-3.13)	.002	2.06 (1.33-3.21)	.001	1.98 (1.27-3.08)	.003
Bottle use >15 mo						
No	1 [Reference]	NA	1 [Reference]	NA	1 [Reference]	NA
Yes	1.24 (0.83-1.84)	.29	1.28 (0.87-1.90)	.21	1.22 (0.82-1.82)	.33
Daily cow’s milk intake						
≤2 cups	1 [Reference]	NA	1 [Reference]	NA	1 [Reference]	NA
>2 cups	1.70 (1.18-2.44)	.005	1.70 (1.19-2.44)	.004	1.68 (1.16-2.42)	.006
Maternal education						
University	1 [Reference]	NA	1 [Reference]	NA	1 [Reference]	NA
College/trades certificate	0.86 (0.47-1.55)	.61	1.07 (0.62-1.86)	.80	1.83 (0.46-1.51)	.54
High school or less	1.13 (0.59-2.17)	.71	1.82 (1.02-3.27)	.04	1.16 (0.60-2.24)	.66
Family income, CAD$^c^						
≥150 000	1 [Reference]	NA	NA	NA	1 [Reference]	NA
80 000-149 999	1.12 (0.76-1.65)	.57	NA	NA	1.14 (0.77-1.67)	.53
40 000-79 999	0.75 (0.41-1.38)	.36	NA	NA	0.82 (0.45-1.51)	.53
<40 000	2.62 (1.45-4.76)	.002	NA	NA	3.08 (1.66-5.72)	<.001
Family at risk for food insecurity						
All other	NA	NA	1 [Reference]	NA	1 [Reference]	NA
Yes	NA	NA	0.57 (0.25-1.30)	.18	0.43 (0.18-1.02)	.06

Abbreviations: BMI, body mass index; CRP, C-reactive protein; OR, odds ratio; NA, not applicable.

^a^ Model 1a was adjusted for age, sex, birth weight, BMI *z *score, CRP level, breastfeeding duration, bottle use, daily cow’s milk intake, infant feeding in the first year of life, and family income. Model 1b was adjusted for age, sex, birth weight, BMI *z *score, CRP level, breastfeeding duration, bottle use, daily cow’s milk intake, infant feeding in the first year of life, and family at risk of food insecurity. Model 2 was adjusted for all variables included in models 1a and 1b.

^b^ Child age was transformed using restricted cubic spline transformation with 3 knots to correspond with the age at scheduled visits (ie, 15, 18, and 24 months).

^c^ To convert Canadian dollars to US dollars, multiply by 0.74.

**Table 5.** Multivariable Logistic Regression Model for the Association of Family Income and Family at Risk of Food Insecurity With Iron Deficiency Anemia^a^

Variable	Model 1a		Model 1b		Model 2	
	OR (95% CI)	*P* Value	OR (95% CI)	*P* Value	OR (95% CI)	*P* Value
Child sex						
Male	1 [Reference]	NA	1 [Reference]	NA	1 [Reference]	NA
Female	0.95 (0.55-1.66)	.86	0.93 (0.54-1.61)	.80	0.94 (0.54-1.63)	.81
Child’s age, mo^b^						
12-15	0.90 (0.58-1.40)	.64	0.91 (0.59-1.41)	.68	0.89 (0.58-1.39)	.61
15-18	1.17 (0.80-1.70)	.42	1.14 (0.79-1.66)	.48	1.18 (0.81-1.72)	.40
18-24	1.05 (0.91-1.20)	.50	1.06 (0.92-1.21)	.38	1.05 (0.92-1.21)	.40
24-29	0.84 (0.51-1.38)	.49	0.83 (0.50-1.35)	.45	0.81 (0.49-1.33)	.43
Birth weight, kg	0.69 (0.44-1.08)	.11	0.67 (0.43-1.04)	.07	0.69 (0.44-1.09)	.11
BMI *z *score	1.15 (0.88-1.51)	.32	1.156 (0.89-1.52)	.28	1.15 (0.88-1.50)	.32
CRP level	0.78 (0.50-1.23)	.29	0.77 (0.49-1.21)	.26	0.78 (0.50-1.22)	.28
Infant feeding first year of life						
Mostly breastfed	1 [Reference]	NA	1 [Reference]	NA	1 [Reference]	NA
Breast milk and formula equally	0.24 (0.09-0.69)	.008	0.30 (0.11-0.81)	.02	0.259 (0.09-0.71)	.009
Mostly formula fed	0.29 (0.09-0.90)	.03	0.35 (0.11-1.06)	.06	0.29 (0.09-0.91)	.04
Breastfeeding duration, mo						
<12	1 [Reference]	NA	1 [Reference]	NA	1 [Reference]	NA
≥12	1.71 (0.81-3.62)	.16	1.78 (0.84-3.75)	.13	1.68 (80-3.55)	.17
Bottle use >15 mo						
No	1 [Reference]	NA	1 [Reference]	NA	1 [Reference]	NA
Yes	1.49 (0.77-2.88)	.24	1.53 (0.79-2.96)	.24	1.44 (0.74-2.81)	.29
Daily cow’s milk intake						
≤2 cups	1 [Reference]	NA	1 [Reference]	NA	1 [Reference]	NA
>2 cups	1.36 (0.73-2.52)	.33	1.32 (0.73-2.49)	.34	1.31 (0.70-2.45)	.40
Maternal education						
University	1 [Reference]	NA	1 [Reference]	NA	1 [Reference]	NA
College/trades certificate	0.56 (0.18-1.71)	.31	0.69 (0.24-2.01)	.50	0.52 (0.17-1.62)	.26
High school or less	2.31 (0.93-5.73)	.07	3.65 (1.69-7.92)	.001	2.40 (0.96-5.99)	.06
Family income, CAD$^c^						
≥150 000	1 [Reference]	NA	NA	NA	1 [Reference]	NA
80 000-149 999	1.40 (0.74-2.68)	.30	NA	NA	1.0 (0.76-2.77)	.26
40 000-79 999	0.94 (0.33-2.68)	.90	NA	NA	1.09 (0.38-3.12)	.88
<40 000	2.55 (0.96-6.75)	.06	NA	NA	3.28 (1.22-8.87)	.02
Family at risk for food insecurity						
All other	NA	NA	1 [Reference]	NA	1 [Reference]	NA
Yes	NA	NA	0.21 (0.03-1.59)	.13	0.16 (0.02-1.23)	.08

Abbreviations: BMI, body mass index; CRP, C-reactive protein; NA, not applicable; OR, odds ratio.

^a^ Model 1a was adjusted for age, sex, birth weight, BMI *z *score, CRP level, breastfeeding duration, bottle use, daily cow’s milk intake, infant feeding in the first year of life, and family income. Model 1b was adjusted for age, sex, birth weight, BMI *z *score, CRP level, breastfeeding duration, bottle use, daily cow’s milk intake, infant feeding in the first year of life, family at risk of food insecurity. Model 2 was adjusted for all variables included in models 1a and 1b.

^b^ Child age was transformed using restricted cubic spline transformation with 3 knots to correspond with the age at scheduled visits (ie, 15, 18, and 24 months).

^c^ To convert Canadian dollars to US dollars, multiply by 0.74.

In the fully adjusted logistic regression model (model 2), there was no association between ID or IDA and risk of FI (ID: OR, 0.43; 95% CI, 0.18-1.02; *P* = .06; IDA: OR, 0.16; 95% CI, 0.02-1.23; *P* = .08) (Table 4 and Table 5). Results from sensitivity analyses were similar to our main findings (eAppendix in the Supplement).

## Discussion

In this large cohort of healthy urban young Canadian children enrolled in primary care settings, we found that a family income of less than CAD $40 000 ($29 534) was associated with 3-fold higher odds of ID and IDA compared with children in the highest family income group. Our analyses included adjusting for important covariates including child factors (ie, age, sex, birth weight, BMI *z *score, CRP level, and maternal education), feeding practices (ie, breastfeeding duration, infant feeding in the first year, cow’s milk intake, and bottle use), and FI status. We found no association of odds of ID or IDA with risk of FI.

Several observational studies from other high-income countries have examined the association between family income and iron status in early childhood.^9,30,31,32^ In the United States, there have been several publications using data from the National Health and Nutrition Examination Survey demonstrating a narrowing of the gap between young children living above and below the federal poverty line in successive survey cycles from 1976 to 2002.^9,31,32^ In Italy, Ferrara et al^54^ assessed iron status in 1250 children aged 8 to 36 months from 1980 to 2010 and found that children from low-income families had a 3-fold to 4-fold increase in ID compared with children from medium- to high-income families.^54^ In New Zealand, Soh et al^55^ assessed iron status in 323 children aged 6 to 24 months and did not identify an association with low family income with adjustment for dietary factors; however, their sample size was smaller.^55^

Results of studies examining the association between FI and iron status in early childhood differ in high-risk vs average-risk populations. Children whose families reported more severe FI were more likely to have IDA,^26,28,29^ but research using US population-level data from the National Health and Nutrition Examination Survey found no association with FI.^30,56^ Our study assessed families at risk for food insecurity, a less severe form representing average risk.

In our study, we found an association between family income and ID but not between risk of FI and ID. Income and FI are closely related but different constructs. Factors that may buffer low-income families from FI include savings and assets, particularly home ownership and income stability^57^; literature on the association between food buying and preparation skills and FI is mixed.^58,59^

We found that having a low family income was associated with an increased risk of ID, even after controlling for important covariates. Children in the lowest income group were more likely to be mostly formula fed in the first year, to drink more than 2 cups of cow’s milk daily, and to have shorter breastfeeding durations. However, even after adjusting for these and other important covariates, including family risk of FI, low income was independently associated with a greater risk of ID. Identifying the causal pathway by which low income leads to ID is beyond the scope of this project, but our results may be related to unmeasured confounders, such as maternal iron status in pregnancy and delayed cord clamping at birth,^60^ both of which are associated with increased iron stores in the first year of life. Our study provides support for the inclusion of family income as an important risk factor in screening for ID in early childhood. Our group has derived and is currently collecting data to externally validate a clinical decision rule to inform targeted screening of young children for ID.^61^

We did not find an association between family at risk of FI and ID, which may be explained by the relative distribution of risk and protective factors. For example, similar to children in the lowest income group, more children from families at risk of FI were mostly formula fed in the first year and had a shorter breastfeeding duration. However, in contrast with the lowest income group, they did not have high cow’s milk consumption, which may have been relatively protective. Our results are consistent with those reported by Metallinos-Katsaras et al,^26^ who also did not find an association between risk of FI and ID, suggesting that this result may be related to iron-rich foods being provided to Women, Infants, and Children–supported families.

Extensive evidence has demonstrated the importance of socioeconomic factors in determining children’s health. The strong association between social determinants in childhood and health throughout the life course in turn is driving increased interest in screening for social needs in clinical care. Multiple health professional bodies have established campaigns encouraging health professionals to screen their patients for unmet social needs.^62,63^ However, many questions remain regarding optimal approaches for identifying and intervening on unmet social needs in health care settings. Dworkin and Garg^64^ advocate for a strength-based, family- and patient-centered approach that emphasizes shared decision-making and respect for family autonomy and priorities and has the capacity to link families to community-based programs and services. However, physicians identify multiple barriers to screening for social needs, including constraints in financial resources and time, misaligned incentives, and physician uncertainty regarding how best to address social needs.^65,66^ Furthermore, there remains a gap in robust studies of appropriate screening instruments and the association of interventions with health outcomes, rather than intermediate process outcomes.^67^

It is unclear whether social needs screening should aim to identify financial strain generally or more specific needs, such as FI. Our study lends support to the approach supported by the College of Family Physicians of Canada, ie, using a general question to identify financial need. (“Do you ever have difficulty making ends meet at the end of the month?”)^68^ In contrast, the American Academy of Pediatrics recommends screening for FI.^69^ Some experts suggest that such an approach may be constrained by parents’ concern regarding the stigma of FI, fear of being judged, and in particular, fear that identifying difficulty with providing adequate food for their children may trigger reports to child welfare authorities and fear of losing their children.^70,71^

Previous work suggests that both policy interventions targeting income security and large-scale food assistance programs can narrow health inequities (including FI).^72^ Canadian studies highlight the association between relatively small changes in income supports for low-income Canadians and improvement in food security status.^73,74^ Unlike the United States, Canada lacks large-scale food assistance programs, such as Women, Infants, and Children or the Supplemental Nutrition Assistance Program, which are associated with reduced but still substantial FI. Currently there is insufficient evidence showing that food programs more effectively reduce FI or improve child health compared with income support programs, suggesting that it is unclear whether public resources should preferentially target income security or food access programs.

### Strengths and Limitations

Strengths of our study include the large sample size of 1245 healthy urban Canadian children recruited in primary care. We used parent-reported family income rather than ecological measures and prospectively collected data on multiple confounders, including child characteristics and feeding practices. The primary outcome, ID, was measured by serum ferritin level, which is considered the best indicator of iron stores.^43^

Our study also had limitations. Our observational study demonstrated important associations with ID and IDA but cannot determine causation. Furthermore, the cross-sectional design precludes conclusions about changes in exposures and outcomes over time. Study participants were recruited from primary care practices in Toronto, Canada, and may not be generalizable to the general population of young children in Canada and other high-income countries. Measures of food insecurity were restricted to brief measures; use of the full HFSSM would have provided a more robust measure. Family income and risk of FI were parent-reported and may be subject to social desirability bias, perhaps particularly for FI reporting. This may have biased the results toward the null. Also, self-reported breastfeeding duration may be subject to recall bias, although this is considered a valid and reliable measure in the first 3 years of life.^44^

## Conclusions

In this study, low family income was associated with an increased risk of ID and IDA in young Canadian children. Risk of FI was not associated with ID or IDA. These findings suggest that targeting income security may be more effective than targeting access to food to reduce health inequities in the prevention of iron deficiency.

## Notes

Supplement. **eBox 1. **Participant Recruitment and Selection of Patients for Inclusion **eBox 2. **Participants Without Blood Tests **eTable. **Characteristics of Children With and Without Blood Testing **eAppendix. **Sensitivity Analyses

## References

[1] Lozoff B, Brittenham GM, Wolf AW, . Iron deficiency anemia and iron therapy effects on infant developmental test performance. Pediatrics. 1987;79(6):981-995. 2438638

[2] Lozoff B, Jimenez E, Wolf AW. Long-term developmental outcome of infants with iron deficiency. N Engl J Med. 1991;325(10):687-694. doi:10.1056/NEJM199109053251004 1870641

[3] Lozoff B, Jimenez E, Hagen J, Mollen E, Wolf AW. Poorer behavioral and developmental outcome more than 10 years after treatment for iron deficiency in infancy. Pediatrics. 2000;105(4):E51. doi:10.1542/peds.105.4.e51 10742372

[4] Lozoff B, Jimenez E, Smith JB. Double burden of iron deficiency in infancy and low socioeconomic status: a longitudinal analysis of cognitive test scores to age 19 years. Arch Pediatr Adolesc Med. 2006;160(11):1108-1113. doi:10.1001/archpedi.160.11.1108 17088512 PMC1866361

[5] Grantham-McGregor S, Ani C. A review of studies on the effect of iron deficiency on cognitive development in children. J Nutr. 2001;131(2S-2):649S-666S. doi:10.1093/jn/131.2.649S 11160596

[6] McCann JC, Ames BN. An overview of evidence for a causal relation between iron deficiency during development and deficits in cognitive or behavioral function. Am J Clin Nutr. 2007;85(4):931-945. doi:10.1093/ajcn/85.4.931 17413089

[7] Oatley H, Borkhoff CM, Chen S, ; TARGet Kids! Collaboration. Screening for iron deficiency in early childhood using serum ferritin in the primary care setting. Pediatrics. 2018;142(6):e20182095. doi:10.1542/peds.2018-2095 30487142

[8] Hartfield D. Iron deficiency is a public health problem in Canadian infants and children. Paediatr Child Health. 2010;15(6):347-350. doi:10.1093/pch/15.6.347 21731416 PMC2921732

[9] Brotanek JM, Gosz J, Weitzman M, Flores G. Secular trends in the prevalence of iron deficiency among US toddlers, 1976-2002. Arch Pediatr Adolesc Med. 2008;162(4):374-381. doi:10.1001/archpedi.162.4.374 18391147

[10] Baker RD, Greer FR; Committee on Nutrition American Academy of Pediatrics. Diagnosis and prevention of iron deficiency and iron-deficiency anemia in infants and young children (0-3 years of age). Pediatrics. 2010;126(5):1040-1050. doi:10.1542/peds.2010-2576 20923825

[11] Boivin M, Hertzman C, Barr RG, . Early Childhood Development: Adverse Experiences and Developmental Health. Royal Society of Canada–Canadian Academy of Health Sciences Expert Panel; 2012.

[12] Pascoe JM, Wood DL, Duffee JH, Kuo A; Committee on Psychosocial Aspects of Child and Family Health; Council on Community Pediatrics. Mediators and adverse effects of child poverty in the United States. Pediatrics. 2016;137(4):e20160340. doi:10.1542/peds.2016-0340 26962239

[13] American Academy of Pediatrics Council on Community Pediatrics. Poverty and child health in the U.S. Pediatrics. 2016;137(4):e2016033. doi:10.1542/peds.2016-0339 26962238

[14] Semega J, Kollar M, Creamer J, Mohanty A. Income and Poverty in the United States: 2018. US Census Bureau; 2019.

[15] Hertzman C. Social geography of developmental health in the early years. Healthc Q. 2010;14(Spec No 1):32-40. doi:10.12927/hcq.2010.21981 20959745

[16] Rose-Jacobs R, Black MM, Casey PH, . Household food insecurity: associations with at-risk infant and toddler development. Pediatrics. 2008;121(1):65-72. doi:10.1542/peds.2006-3717 18166558

[17] Food and Agriculture Organization of the United Nations. Trade reforms and food security: conceptualizing the linkages. Accessed July 2, 2020. http://www.fao.org/3/y4671e/y4671e06.htm

[18] Coleman-Jensen A, Rabbitt MP, Gregory CA, Singh A. Household Food Security in the United States in 2017. US Department of Agriculture, Economic Research Service; 2018.

[19] Ryu JH, Bartfeld JS. Household food insecurity during childhood and subsequent health status: the early childhood longitudinal study—kindergarten cohort. Am J Public Health. 2012;102(11):e50-e55. doi:10.2105/AJPH.2012.300971 22994255 PMC3477974

[20] Cook JT, Black M, Chilton M, . Are food insecurity’s health impacts underestimated in the U.S. population? marginal food security also predicts adverse health outcomes in young U.S. children and mothers. Adv Nutr. 2013;4(1):51-61. doi:10.3945/an.112.003228 23319123 PMC3648739

[21] Hernandez DC, Jacknowitz A. Transient, but not persistent, adult food insecurity influences toddler development. J Nutr. 2009;139(8):1517-1524. doi:10.3945/jn.109.105593 19535426

[22] Melchior M, Chastang JF, Falissard B, . Food insecurity and children’s mental health: a prospective birth cohort study. PLoS One. 2012;7(12):e52615-e52615. doi:10.1371/journal.pone.0052615 23300723 PMC3530436

[23] Kirkpatrick SI, McIntyre L, Potestio ML. Child hunger and long-term adverse consequences for health. Arch Pediatr Adolesc Med. 2010;164(8):754-762. doi:10.1001/archpediatrics.2010.117 20679167

[24] Whitaker RC, Phillips SM, Orzol SM. Food insecurity and the risks of depression and anxiety in mothers and behavior problems in their preschool-aged children. Pediatrics. 2006;118(3):e859-e868. doi:10.1542/peds.2006-0239 16950971

[25] Council on Community Pediatrics; Committee on Nutrition. Promoting food security for all children. Pediatrics. 2015;136(5):e1431-e1438. doi:10.1542/peds.2015-3301 26498462

[26] Metallinos-Katsaras E, Colchamiro R, Edelstein S, Siu E. Household food security status is associated with anemia risk at age 18 months among low-income infants in Massachusetts. J Acad Nutr Diet. 2016;116(11):1760-1766. doi:10.1016/j.jand.2016.06.008 27451132

[27] Pirkle CM, Lucas M, Dallaire R, . Food insecurity and nutritional biomarkers in relation to stature in Inuit children from Nunavik. Can J Public Health. 2014;105(4):e233-e238. doi:10.17269/cjph.105.4520 25166123 PMC6972159

[28] Park K, Kersey M, Geppert J, Story M, Cutts D, Himes JH. Household food insecurity is a risk factor for iron-deficiency anaemia in a multi-ethnic, low-income sample of infants and toddlers. Public Health Nutr. 2009;12(11):2120-2128. doi:10.1017/S1368980009005540 19405987

[29] Skalicky A, Meyers AF, Adams WG, Yang Z, Cook JT, Frank DA. Child food insecurity and iron deficiency anemia in low-income infants and toddlers in the United States. Matern Child Health J. 2006;10(2):177-185. doi:10.1007/s10995-005-0036-0 16328705

[30] Alaimo K, Olson CM, Frongillo EA Jr, Briefel RR. Food insufficiency, family income, and health in US preschool and school-aged children. Am J Public Health. 2001;91(5):781-786. doi:10.2105/AJPH.91.5.781 11344887 PMC1446676

[31] Brotanek JM, Halterman JS, Auinger P, Flores G, Weitzman M. Iron deficiency, prolonged bottle-feeding, and racial/ethnic disparities in young children. Arch Pediatr Adolesc Med. 2005;159(11):1038-1042. doi:10.1001/archpedi.159.11.1038 16275794

[32] Brotanek JM, Gosz J, Weitzman M, Flores G. Iron deficiency in early childhood in the United States: risk factors and racial/ethnic disparities. Pediatrics. 2007;120(3):568-575. doi:10.1542/peds.2007-0572 17766530

[33] Carsley S, Borkhoff CM, Maguire JL, ; TARGet Kids! Collaboration. Cohort profile: The Applied Research Group for Kids (TARGet Kids!). Int J Epidemiol. 2015;44(3):776-788. doi:10.1093/ije/dyu123 24982016 PMC4521122

[34] Randall Simpson J, Gumbley J, Whyte K, . Development, reliability, and validity testing of Toddler NutriSTEP: a nutrition risk screening questionnaire for children 18-35 months of age. Appl Physiol Nutr Metab. 2015;40(9):877-886. doi:10.1139/apnm-2015-0048 26300014

[35] Parkin PC, Hamid J, Borkhoff CM, . Laboratory reference intervals in the assessment of iron status in young children. BMJ Paediatr Open. 2017;1(1):e000074. doi:10.1136/bmjpo-2017-000074 29637115 PMC5862219

[36] Namaste SM, Rohner F, Huang J, . Adjusting ferritin concentrations for inflammation: Biomarkers Reflecting Inflammation and Nutritional Determinants of Anemia (BRINDA) project. Am J Clin Nutr. 2017;106(suppl 1):359S-371S. 28615259 10.3945/ajcn.116.141762 PMC5490647

[37] Statistics Canada. Median total income, by family type, by census metropolitan area. Accessed September 19, 2017. https://www.statcan.gc.ca/tables-tableaux/sum-som/101/cst01/famil107a-eng.htm

[38] Children’s Health Watch. Accessed December 5, 2019. https://childrenshealthwatch.org/public-policy/hunger-vital-sign/

[39] US Department of Agriculture. US Household Food Security Survey Module: three stage design, with screeners. Accessed July 2, 2002. https://www.ers.usda.gov/media/8271/hh2012.pdf

[40] Hager ER, Quigg AM, Black MM, . Development and validity of a 2-item screen to identify families at risk for food insecurity. Pediatrics. 2010;126(1):e26-e32. doi:10.1542/peds.2009-3146 20595453

[41] Borkhoff CM, Bayoumi I, Nurse KM, . A single NutriSTEP question as a food insecurity screening tool. Paediatr Child Health. 2016;21(5):274. Accessed July 15, 2020. https://cps.multilearning.com/cps/2016/eposter/128202/cory.borkhoff.a.single.nutristep.question.as.a.food.insecurity.screening.tool.html?f=media=1

[42] World Health Organization. Serum ferritin concentrations for assessment of iron status and iron deficiency in populations. Accessed June 27, 2019. https://www.who.int/vmnis/indicators/serum_ferritin.pdf

[43] Zimmermann MB. Methods to assess iron and iodine status. Br J Nutr. 2008;99(S3)(suppl 3):S2-S9. doi:10.1017/S000711450800679X 18598585

[44] Li R, Scanlon KS, Serdula MK. The validity and reliability of maternal recall of breastfeeding practice. Nutr Rev. 2005;63(4):103-110. doi:10.1111/j.1753-4887.2005.tb00128.x 15869124

[45] Sypes EE, Parkin PC, Birken CS, ; TARGet Kids! Collaboration. Higher body mass index is associated with iron deficiency in children 1 to 3 years of age. J Pediatr. 2019;207:198-204.e1. doi:10.1016/j.jpeds.2018.11.035 30630632

[46] Maguire JL, Lebovic G, Kandasamy S, ; TARGet Kids! Collaboration. The relationship between cow’s milk and stores of vitamin D and iron in early childhood. Pediatrics. 2013;131(1):e144-e151. doi:10.1542/peds.2012-1793 23248224

[47] Maguire JL, Salehi L, Birken CS, ; TARGet Kids! Collaboration. Association between total duration of breastfeeding and iron deficiency. Pediatrics. 2013;131(5):e1530-e1537. doi:10.1542/peds.2012-2465 23589818

[48] Sutcliffe TL, Khambalia A, Westergard S, Jacobson S, Peer M, Parkin PC. Iron depletion is associated with daytime bottle-feeding in the second and third years of life. Arch Pediatr Adolesc Med. 2006;160(11):1114-1120. doi:10.1001/archpedi.160.11.1114 17088513

[49] Harrell F. Regression Modeling Strategies With Applications to Linear Models, Logistic Regression, and Survival Analysis. Springer;2001. doi:10.1007/978-1-4757-3462-1

[50] O’Brien RM. A caution regarding rules of thumb for variance inflation factors. Qual Quant. 2007;(41):673-690. doi:10.1007/s11135-006-9018-6

[51] van Buuren S, Groothuis-Oudshoorn K. Mice: multivariate imputation by chained equations in R. J Stat Softw. 2011;45(3):1-67. doi:10.18637/jss.v045.i03

[52] Little R, Rubin DB. Statistical Analysis With Missing Data. Wiley; 2002. doi:10.1002/9781119013563

[53] Hayes-Larson E, Kezios KL, Mooney SJ, Lovasi G. Who is in this study, anyway? guidelines for a useful Table 1. J Clin Epidemiol. 2019;114:125-132. doi:10.1016/j.jclinepi.2019.06.011 31229583 PMC6773463

[54] Ferraro S, Panteghini M. Is serum human epididymis protein 4 ready for prime time? Ann Clin Biochem. 2014;51(Pt 2):128-136. doi:10.1177/0004563213500657 24031041

[55] Soh P, Ferguson EL, McKenzie JE, Homs MYV, Gibson RS. Iron deficiency and risk factors for lower iron stores in 6-24-month-old New Zealanders. Eur J Clin Nutr. 2004;58(1):71-79. doi:10.1038/sj.ejcn.1601751 14679370

[56] Eicher-Miller HA, Mason AC, Weaver CM, McCabe GP, Boushey CJ. Food insecurity is associated with iron deficiency anemia in US adolescents. Am J Clin Nutr. 2009;90(5):1358-1371. doi:10.3945/ajcn.2009.27886 19776137

[57] Tarasuk V, Fafard St-Germain A-A, Mitchell A. Geographic and socio-demographic predictors of household food insecurity in Canada, 2011-12. BMC Public Health. 2019;19(1):12. doi:10.1186/s12889-018-6344-2 30606152 PMC6318847

[58] Huisken A, Orr SK, Tarasuk V. Adults’ food skills and use of gardens are not associated with household food insecurity in Canada. Can J Public Health. 2017;107(6):e526-e532. doi:10.17269/CJPH.107.5692 28252370 PMC6972328

[59] Eicher-Miller HA, Mason AC, Abbott AR, McCabe GP, Boushey CJ. The effect of Food Stamp Nutrition Education on the food insecurity of low-income women participants. J Nutr Educ Behav. 2009;41(3):161-168. doi:10.1016/j.jneb.2008.06.004 19411049

[60] Andersson O, Hellström-Westas L, Andersson D, Domellöf M. Effect of delayed versus early umbilical cord clamping on neonatal outcomes and iron status at 4 months: a randomised controlled trial. BMJ. 2011;343:d7157. doi:10.1136/bmj.d7157 22089242 PMC3217058

[61] Borkhoff CM, Parkin PC, Persaud N, . A clinical decision rule to identify young asymptomatic children at risk for iron deficiency. CIHR Early Career Investigator Grant 2019-2022. Accessed March 26, 2020. https://cihr-irsc.gc.ca/e/51427.html

[62] American Academy of Family Physicians. Social determinants of health policy. Accessed Nov 18 2019. https://www.aafp.org/about/policies/all/social-determinants.html

[63] Hagan JF, Shaw JS, Duncan MD, eds. Bright Futures: Guidelines for Health Supervision of Infants, Children, and Adolescents. 4th Ed. American Academy of Pediatrics; 2017.

[64] Dworkin PH, Garg A. Considering approaches to screening for social determinants of health. Pediatrics. 2019;144(4):e20192395. doi:10.1542/peds.2019-2395 31548336

[65] Schickedanz A, Hamity C, Rogers A, Sharp AL, Jackson A. Clinician experiences and attitudes regarding screening for social determinants of health in a large integrated health system. Med Care. 2019;57(Suppl 6 2):S197-S201. doi:10.1097/MLR.0000000000001051 31095061 PMC6721844

[66] Bayoumi I, Coo H, Purkey E, . Implementing a clinical tool to screen for poverty in primary and pediatric care settings. Paper presented at: North American Primary Care Research Group; November 18, 2017; Montreal, Canada.

[67] Davidson KW, McGinn T. Screening for social determinants of health: the known and unknown. JAMA. 2019;322(11):1037-1038. doi:10.1001/jama.2019.10915 31465095

[68] College of Family Physicians of Canada. Poverty: a clinical tool for primary care providers. Accessed July 1, 2020. https://www.cfpc.ca/Poverty_Tools/

[69] Council on Community Pediatrics; Committee on Nutrition. Promoting food security for all children. Pediatrics. 2015;136(5):e1431-e1438. doi:10.1542/peds.2015-3301 26498462

[70] Barnidge E, Krupsky K, LaBarge G, Arthur J. Food insecurity screening in pediatric clinical settings: a caregivers’ perspective. Matern Child Health J. 2020;24(1):101-109. 31494801 10.1007/s10995-019-02785-6

[71] Knowles M, Khan S, Palakshappa D, . Successes, challenges, and considerations for integrating referral into food insecurity screening in pediatric settings. J Health Care Poor Underserved. 2018;29(1):181-191. doi:10.1353/hpu.2018.0012 29503293

[72] Loopstra R. Interventions to address household food insecurity in high-income countries. Proc Nutr Soc. 2018;77(3):270-281. doi:10.1017/S002966511800006X 29580316

[73] Loopstra R, Tarasuk V. Severity of household food insecurity is sensitive to change in household income and employment status among low-income families. J Nutr. 2013;143(8):1316-1323. doi:10.3945/jn.113.175414 23761648

[74] Li N, Dachner N, Tarasuk V. The impact of changes in social policies on household food insecurity in British Columbia, 2005-2012. Prev Med. 2016;93:151-158. doi:10.1016/j.ypmed.2016.10.002 27729259

