# Supplementary material for: Association of Family Income and Risk of Food Insecurity With Iron Status in Young Children
Source: JAMA Netw Open. 2020 Jul 30;3(7):e208603. doi: 10.1001/jamanetworkopen.2020.8603 (PMC12578488; doi:10.1001/jamanetworkopen.2020.8603)
Supplement: Supplement. — eBox 1. Participant Recruitment and Selection of Patients for Inclusion eBox 2. Participants Without Blood Tests eTable. Characteristics of Children With and Without Blood Testing eAppendix. Sensitivity Analyses [file jamanetwopen-e208603-s001.pdf]

Supplementary Online Content

Bayoumi I, Parkin PC, Birken CS, Maguire JL, Borkhoff CM; TARGet Kids! Collaboration. Association of family income and risk of food insecurity with iron status in young children. *JAMA Netw Open*. 2020;3(7):e208603. doi:10.1001/jamanetworkopen.2020.8603

eBox 1. Participant Recruitment and Selection of Patients for Inclusion

eBox 2. Participants Without Blood Tests

eTable. Characteristics of Children With and Without Blood Testing

eAppendix. Sensitivity Analyses

This supplementary material has been provided by the authors to give readers additional information about their work.

© 2020 Bayoumi I et al. *JAMA Network Open.*

eBox 1. Participant Recruitment and Selection of Patients for Inclusion (*n*=1245)

Characteristic	No.
Consent obtained from parents of healthy children between ages 12-29 months enrolled in the TARGet Kids! cohort	4571
Exclusion criteria	
No self-report family income data	255
No household food security data	222
No serum ferritin data	2170
Serum ferritin value > 200 µg/L	1
CRP > 5 mg/L or missing	98
Receiving iron supplementation	21
Receiving multivitamins with iron	19
Receiving both iron supplementation and multivitamins with iron	7
Missing iron supplementation data	56
Final cohort	1245

© 2020 Bayoumi I et al. *JAMA Network Open.*

eBox 2. Participants Without Blood Tests (*n*=2074)

Characteristic	No.
Consent obtained from parents of healthy children between ages 12-29 months enrolled in the TARGet Kids! cohort	4571
Exclusion criteria	
No self-report family income data	646
No household food security data	87
With serum ferritin data	1638
Receiving iron supplementation OR multivitamins with iron	67
Missing iron supplementation data	59
Final cohort	2074

© 2020 Bayoumi I et al. *JAMA Network Open.*

eTable. Characteristics of Children With and Without Blood Testing

**Characteristic**	**With Blood Testing**		**Without Blood Testing**	
	n	Median (IQR) or N (%)	n	Median (IQR) or N (%)
**Patient-level characteristics**				
Female sex	1245	595 (47.8)	2074	945 (45.6)
Child's age, months	1245	18.1 (13.3–24.0)	2074	16.0 (12.4–18.8)
Family income (CAN $)	1245		2074	
$150,000 +		517 (41.5)		895 (43.2)
$80,000 - $149,999		435 (34.9)		680 (32.8)
$40,000 - $79,999		162 (13.0)		284 (13.7)
Less than $40,000		131 (10.5)		215 (10.4)
Family at risk for food insecurity	1245		2074	
All other		1168 (93.8)		1922 (92.7)
Yes		77 (6.2)		152 (7.3)
**Laboratory characteristics**				
Serum ferritin (µg/L)	1245	23 (15, 35)	2074	-
Iron deficiency, yes	1245	185 (14.9)	2074	-

Data regarding baseline characteristics are presented as median (IQR) or N (%).

© 2020 Bayoumi I et al. *JAMA Network Open.*

eAppendix. Sensitivity Analyses

Tables 3A and 3B. Multivariable logistic regression model for the association between income and family at risk for FI on ID (OR; 95% CI)[1]

**Variable**	**Model 1a**		**Model 1b**		**Model 2**	
	**OR (95% CI)**	***P* Value**	**OR (95% CI)**	***P* Value**	**OR (95% CI)**	***P* Value**
**Table 3A.** Multivariable logistic regression model for the association between income and family at risk for food insecurity on ID (OR and 95% CI)[1]						
Note – Family at risk for FI is based on 3 categories: Family at risk for FI is defined as an affirmative response to at least 1 of the 2 items of the HFSS or "Sometimes" or Most of the time" to the 1-item NutriSTEP; Family at risk for FI is defined by a response "Rarely" to the 1-item NutriSTEP.						
Family Income (CAN $)						
$150,000 +	1.00				1.00	
$80,000 - $149,999	1.12 (0.76, 1.65)	.57			1.14 (0.77, 1.67)	.52
$40,000 - $79,999	0.75 (0.41, 1.38)	.36			0.82 (0.45, 1.51)	.53
Less than $40,000	2.62 (1.45, 4.76)	.002			3.08 (1.66, 5.73)	<.001
Family at risk for food insecurity						
Food secure n=1047			1.00		1.00	
Family *rarely* at risk for FI n=121			1.01 (0.59, 1.74)	.96	1.00 (0.58, 1.73)	0.99
Family at risk for FI n=77			0.57 (0.25, 1.31)	.18	0.43 (0.18, 1.02)	0.06
**Table 3B.** Multivariable logistic regression model for the association between income and family at risk for food insecurity on ID (OR and 95% CI)[1]						
Note - Food security is defined as an affirmative response to at least 1 of the 2 items of the HFSS or any response other than "Never" to the 1-item NutriSTEP.						
Family Income (CAN $)						
$150,000 +	1.00				1.00	
$80,000 - $149,999	1.12 (0.76, 1.65)	.57			1.14 (0.78, 1.69)	.50
$40,000 - $79,999	0.75 (0.41, 1.38)	.36			0.80 (0.44, 1.47)	.48
Less than $40,000	2.62 (1.45, 4.76)	.002			2.81 (1.53, 5.18)	<.001
Family at risk for food insecurity						
All other n=1047			1.00		1.00	
Yes n=198			0.84 (0.53, 1.34)	.47	0.77 (0.47, 1.25)	.28

OR indicates odds ratio; CI indicates confidence interval; zBMI indicates body mass index z score.

[1]Model 1a - adjusted for age, sex, birthweight, zBMI, maternal education, CRP, breastfeeding duration, bottle use, daily cow's milk intake, infant feeding in the 1st year of life, family income. Model 1b – adjusted for age, sex, birthweight, zBMI, maternal education, CRP, breastfeeding duration, bottle use, daily cow's milk intake, infant feeding in the 1st year of life, family at risk for food security. Model 2 – adjusted for all variables included in Models 1a and 1b.

[2]Child age was transformed using restricted cubic spline transformation with 3 knots to correspond to the age at scheduled visits (15, 18, and 24 months).

© 2020 Bayoumi I et al. *JAMA Network Open.*

Supplementary Tables 4A and 4B. Multivariable logistic regression model for the association between income and family at risk for food insecurity on IDA (OR and 95% CI)[1]

Variable	Model 1a		Model 1b		Model 2	
	**OR (95% CI)**	***P* Value**	**OR (95% CI)**	***P* Value**	**OR (95% CI)**	***P* Value**
**Table 4A.** Multivariable logistic regression model for the association between income and family at risk for food insecurity on IDA[1]						
Note – Family at risk for FI is based on 3 categories: Family at risk for FI is defined as an affirmative response to at least 1 of the 2 items of the HFSS or “Sometimes” or Most of the time” to the 1-item NutriSTEP; Family at risk for FI is defined by a response “Rarely” to the 1-item NutriSTEP.						
Family income (CAN $)						
$150,000 +	1.00				1.00	
$80,000 - $149,999	1.40 (0.74, 2.68)	.30			1.49 (0.78, 2.85)	.23
$40,000 - $79,999	0.94 (0.33, 2.68)	.90			1.16 (0.40, 3.36)	.78
Less than $40,000	2.55 (0.96, 6.75)	.06			3.41 (1.26, 9.26)	.02
Family at risk for food insecurity						
Food secure n=909			1.00		1.00	
Family *rarely* at risk for FI n=110			0.68 (0.26 1.80)	.44	0.63 (0.24, 1.69)	.36
Family at risk for FI n=71			0.20 (0.03, 1.53)	.12	0.15 (0.02, 1.15)	.07
**Table 4B.** Multivariable logistic regression model for the association between income and family at risk for food insecurity on IDA[1]						
Note - Food security is defined as an affirmative response to at least 1 of the 2 items of the HFSS or any response other than “Never” to the 1-item NutriSTEP.						
Family income (CAN $)						
$150,000 +	1.00				1.00	
$80,000 - $149,999	1.40 (0.74, 2.68)	.30			1.50 (0.78, 2.87)	.22
$40,000 - $79,999	0.94 (0.33, 2.68)	.90			1.14 (0.39, 3.30)	.81
Less than $40,000	2.55 (0.96, 6.75)	.06			3.13 (1.16, 8.44)	.02
Family at risk for food insecurity						
All other n=909			1.00		1.00	
Yes n=181			0.49 (0.20, 1.20)	0.12	0.42 (0.17, 1.05)	.06

OR indicates odds ratio; CI indicates confidence interval; zBMI indicates body mass index z score.

[1]Model 1a - adjusted for age, sex, birthweight, zBMI, maternal education, CRP, breastfeeding duration, bottle use, daily cow’s milk intake, infant feeding in the 1st year of life, family income. Model 1b – adjusted for age, sex, birthweight, zBMI, maternal education, CRP, breastfeeding duration, bottle use, daily cow’s milk intake, infant feeding in the 1st year of life, family at risk for food security. Model 2 – adjusted for all variables included in Models 1a and 1b.

[2]Child age was transformed using restricted cubic spline transformation with 3 knots to correspond to the age at scheduled visits (15, 18, and 24 months).

© 2020 Bayoumi I et al. *JAMA Network Open.*

© 2020 Bayoumi I et al. *JAMA Network Open.*
